# School closures during the 2009 influenza pandemic: national and local experiences

**DOI:** 10.1186/1471-2334-14-207

**Published:** 2014-04-16

**Authors:** Simon Cauchemez, Maria D Van Kerkhove, Brett N Archer, Martin Cetron, Benjamin J Cowling, Peter Grove, Darren Hunt, Mira Kojouharova, Predrag Kon, Kumnuan Ungchusak, Hitoshi Oshitani, Andrea Pugliese, Caterina Rizzo, Guillaume Saour, Tomimase Sunagawa, Amra Uzicanin, Claude Wachtel, Isaac Weisfuse, Hongjie Yu, Angus Nicoll

**Affiliations:** 1 Department of Infectious Disease Epidemiology, MRC Centre for Outbreak Analysis and Modelling, School of Public Health, Imperial College, London, UK; 2 Mathematical Modelling of Infectious Diseases Unit, Institut Pasteur, 28 rue du Dr Roux, 75724 Paris, Cedex 15, France; 3 National Institute for Communicable Diseases, National Health Laboratory Service, Johannesburg, South Africa; 4 Global Migration and Quarantine, National Center for Emerging and Zoonotic Diseases, Centers for Disease Control and Prevention, Atlanta, USA; 5 School of Public Health, Li Ka Shing Faculty of Medicine, The University of Hong Kong, Hong Kong Special Administrative Region, China; 6 Department of Health, London, UK; 7 Office of the Director of Public Health, New Zealand Ministry of Health, Wellington, New Zealand; 8 National Centre of Infectious and Parasitic Diseases, Sofia, Bulgaria; 9 Center for Disease Control and Prevention, City Institute of Public Health Belgrade, Belgrade, Serbia; 10 Department of Disease Control, Ministry of Public Health, Nonthaburi, Thailand; 11 Department of Virology, Tohoku University Graduate School of Medicine, Tokyo, Japan; 12 Dipartimento di Matematica, Università di Trento, Trento, Italy; 13 National Center for Epidemiology, Surveillance and Health Promotion, Istituto Superiore di Sanità, Roma, Italy; 14 Ministère de l’intérieur, Paris, France; 15 Infectious Disease Surveillance Center, National Institute of Infectious Diseases, Sendai, Japan; 16 Secretariat général de la défense et de la sécurite nationale – Prime minister Office, Paris, France; 17 Department of Health and Mental Hygiene, New York City, USA; 18 Division for Infectious Disease Control and Prevention, Chinese Center for Disease Control and Prevention, Beijing, China; 19 Influenza coordination, European Centre for Disease Prevention and Control, Stockholm, Sweden

## Abstract

**Background:**

School closure is a non-pharmaceutical intervention that was considered in many national pandemic plans developed prior to the start of the influenza A(H1N1)pdm09 pandemic, and received considerable attention during the event. Here, we retrospectively review and compare national and local experiences with school closures in several countries during the A(H1N1)pdm09 pandemic. Our intention is not to make a systematic review of country experiences; rather, it is to present the diversity of school closure experiences and provide examples from national and local perspectives.

**Methods:**

Data were gathered during and following a meeting, organized by the European Centres for Disease Control, on school closures held in October 2010 in Stockholm, Sweden. A standard data collection form was developed and sent to all participants. The twelve participating countries and administrative regions (Bulgaria, China, France, Hong Kong Special Administrative Region (SAR), Italy, Japan, New Zealand, Serbia, South Africa, Thailand, United Kingdom, and United States) provided data.

**Results:**

Our review highlights the very diverse national and local experiences on school closures during the A(H1N1)pdm09 pandemic. The processes including who was in charge of making recommendations and who was in charge of making the decision to close, the school-based control strategies, the extent of school closures, the public health tradition of responses and expectations on school closure varied greatly between countries. Our review also discusses the many challenges associated with the implementation of this intervention and makes recommendations for further practical work in this area.

**Conclusions:**

The single most important factor to explain differences observed between countries may have been the different public health practises and public expectations concerning school closures and influenza in the selected countries.

## Background

The use of school closures during influenza epidemics and pandemics as a non-pharmaceutical intervention (NPI) is a topic that has received considerable attention from policy makers, the public health research community, the public and the media. This was particularly true during the 2009 H1N1 influenza (A(H1N1)pdm09) pandemic [1]. School closure was also extensively considered in national pandemic plans developed prior to the start of the pandemic [2-6] and during previous pandemics of the 20^th^ century [7-11].

Prior to the 2009 pandemic, a multidisciplinary perspective was used at a workshop organised under the European Union French Presidency (2008) to review the various aspects of school closures as a public health measure [12]. That review noted how the severity and impact of each pandemic is different and that the impact and relevance of school closure would, to a large extent, depend on the epidemiological and virologic characteristics of the pandemic strain, and the severity of disease [12]. For example, school closure may have had a more substantial effect during the 1957 pandemic, when much of the transmission occurred among children, than it would have had in 1918 when young adults were also affected, or in 1968 when illness attack rates were similar among children and adults [12].

The review also highlighted that the generic expression “school closure” reflects very different strategies. School closure could be reactive (i.e. when children or staff of the school start experiencing illness) or proactive (i.e. before substantial transmission in the school); the duration could vary from a few days to a few months; and include all children and staff (“school closure”) or specific classes with the remainder of the school remaining open (“class dismissal”). At the time, the review concluded that health benefits could be expected (in particular a reduction of healthcare service demand at the peak of the outbreak) to an extent that would depend on the epidemiological characteristics of the virus and the way the policy would be implemented. Equally though, it was recognised that school closure is associated with high economic, social and educational costs and could potentially disrupt healthcare provision via increased absenteeism of clinical staff attending to their children [12]. Since then, analyses of additional data collected during the A(H1N1)pdm09 pandemic have made it possible to further quantify the impact of closures so that literature assessing impact is now substantial [7-11,13-25].

Although essential, assessment of impact is only one of the elements that inform school closure policies. Indeed, national policy makers are constrained by the structure of their political and school systems as well as the local perspective/culture on health issues. Paradoxically, those factors as well as many simple yet essential questions on school closure during the A(H1N1)pdm09 pandemic remain poorly documented. Were schools closed during the A(H1N1)pdm09 pandemic around the world? If so, how and to what extent? What were the decision processes and how was the intervention perceived? What were the operational issues associated with school closure? Why is it that certain countries implemented large scale closure policies while others did not recommend the use of school closure as a mitigation policy? To address these questions, we first position school closures in the context of the A(H1N1)pdm09 pandemic. We then retrospectively review and compare national and local experiences with school closures in several countries during the A(H1N1)pdm09 pandemic. Finally, we discuss lessons learnt.

## Methods

Here we review the experiences of school closures during the A(H1N1)pdm09 pandemic and for seasonal influenza for eleven countries and one administrative region that had prepared pandemic plans at a national or local level. The data used in this review were obtained during and following a meeting, organized by the European Centres for Disease Prevention and Control (ECDC), on school closures held in October 2010 in Stockholm, Sweden. At the meeting, local and national experiences were presented from six countries and one administrative region by country representatives from local and national institutions involved in or providing input into school closure policies: Bulgaria, the United Kingdom (UK), France, Hong Kong SAR (HK), Italy, Japan and the United States (US; national and New York City). Following the meeting, SC, MVK and AN contacted country representatives of five additional countries (China, New Zealand, Serbia, South Africa and Thailand) to contribute data and information on their country’s experiences of school closure. All the country representatives are listed in the author list of the paper.

Our intention was not to make a systematic review of all national and local experiences. Rather, it was to describe the diversity of school closure experiences and provide examples from national and local perspectives. As a consequence, the participating countries and administrative regions (Bulgaria, China, France, HK, Italy, Japan, New Zealand, Serbia, South Africa, Thailand, UK, US) included in this review were known to represent a range of responses but are not a representative sample of countries around the world. The decision to invite countries to the initial ECDC meeting in Stockholm was made on the same basis.

A standard data collection form was used for data collection and sent to study participants (see Additional file 1). The data collection form contained questions about school closure during seasonal influenza epidemics and in pre-pandemic plans, recommendations, decision making and extent of school closure during the A(H1N1)pdm09 pandemic. Data were summarized in tables and figures. For each location, the number of schools affected by closures per million inhabitants was computed. There was no need to consult an ethics committee as no data on individuals were collected.

## Results

### The epidemiology of the pandemic and use of school closures

Early in the A(H1N1)pdm09 pandemic, it was clear from early data from the US, Mexico and the UK that transmission was heavily focused in children [26-29], which was later confirmed from epidemiologic data from other countries as they became affected. Initial media reports from Mexico in early April 2009 caused concerns about the severity of the emerging pandemic. While the severity of the pandemic was difficult to assess early on, a very severe scenario (e.g., mortality similar to what was reported during the 1918 pandemic) was quickly excluded [27,30]. More precise assessment of severity was harder to make and it was appreciated that severity was indeed a complicated concept.

**Figure 1:**
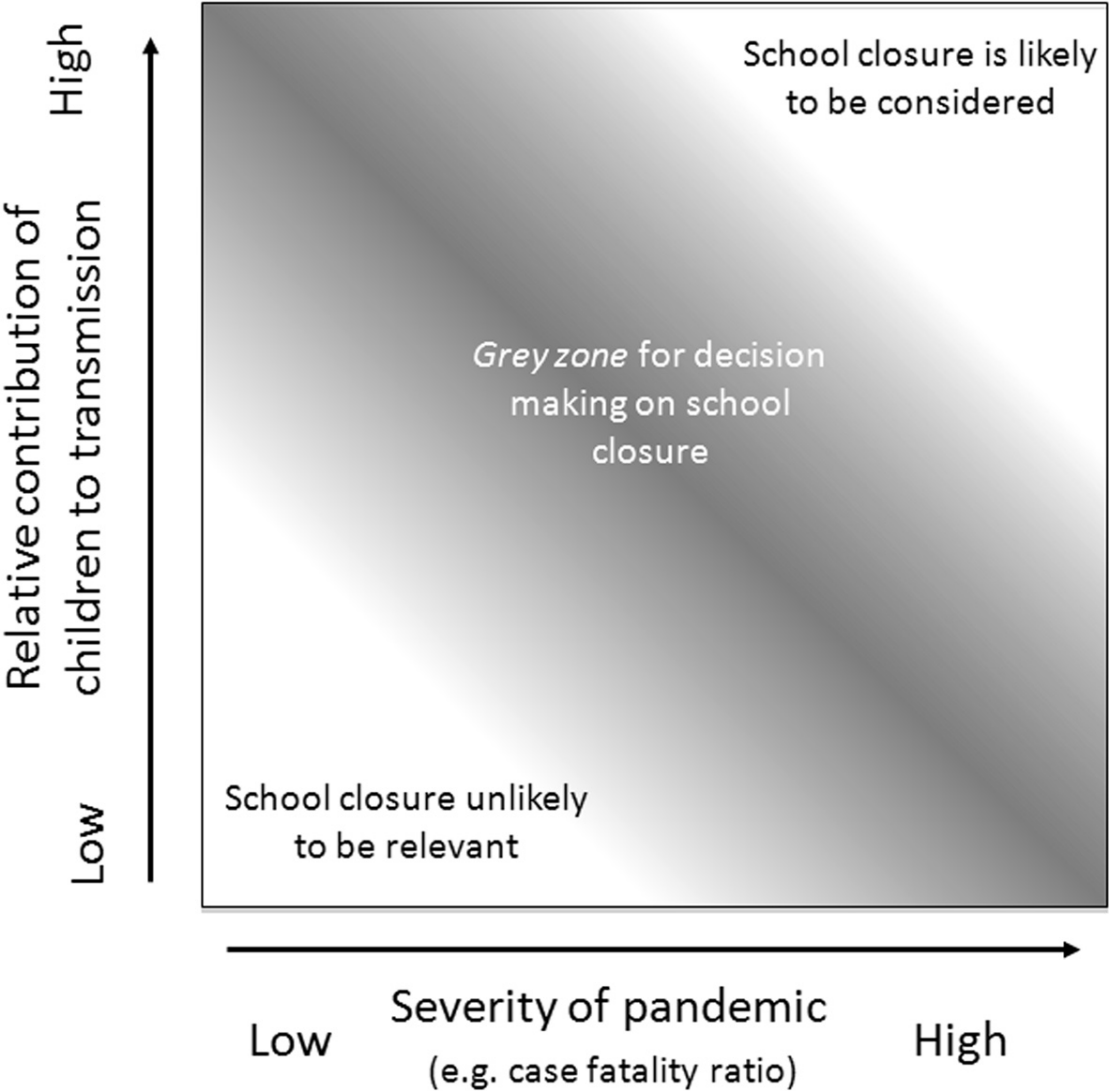
Epidemiological characteristics and relevance of school closures.

Those epidemiological characteristics of relatively low (yet uncertain) severity and high transmission (especially in children) meant that the A(H1N1)pdm09 pandemic fell within the ambiguous or *“grey”* zone for scientific advisers and decision makers (Figure 1). While closing schools during the A(H1N1)pdm09 pandemic was expected to have an impact on transmission because of the age-specific immunity profile, it was unclear whether the potential benefits were worth the high economic and social costs. The absence of clear answers to this and other questions, the complexity of decision making and school systems left fertile ground for divergent views and interpretations on the relevance of school closures in the 2009 context. This was partly reflected by the heterogeneity in the policy options taken by the different countries.

### School closures during seasonal influenza outbreaks and other public health crises

**Figure 2:**
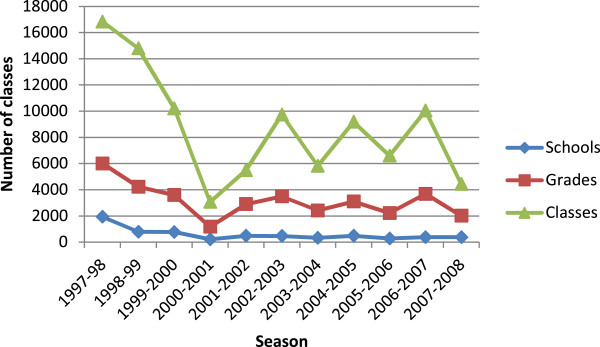
**Number of classes that were affected by school, grade or class closures in Japan during seasonal influenza epidemics between 1997 and 2008. (Source: **http://idsc.nih.go.jp/idwr/kanja/infreport/report.html**).**

Closing schools during seasonal influenza epidemics is a standard policy in two of the 12 countries and administrative region that participated in this review (Japan and Bulgaria). Japan implements a policy of closure of classes, grades and schools (C-CGS) during seasonal influenza epidemics. This is a gradualist policy. For example, Japan will close a class if a certain percentage (usually 10-20%) of students are absent; close a grade if ≥ 2 classes in the grade meet the above criterion; and close a school if ≥ 2 grades of the school meet the same criterion. The exact criteria for closures are usually defined by the local board of education; but a final decision is made by each school. In Japan, there is no nationwide recommendation apart from the notification to schools from the Ministry of Education (Item No. 1125) about the prevention of influenza-like illness (ILI) that dates back to 1982 and indicates that “class closure should be considered when the rate of student absenteeism due to infection reaches approximately 15-20%”. This notification from 1982 shows the long history of the C-CGS policy in the country and references to the policy can be found in reports dating back to the 1957 pandemic. Figure 2 shows the extent to which the C-CGS policy was implemented during seasonal influenza epidemics between 1997 and 2008. In that period, on average, 8,746 classes were closed each year. 3,166 and 594 classes affected by grade and school closures, respectively. The average proportion of classes affected by full school closures was low (5%).

Bulgaria also has a long history of closing schools during seasonal influenza epidemics. Such policies were first recommended in the 1970s. If more than 30% of schoolchildren are absent because of illness, a temporary school closure of individual schools or of all schools in the region may be considered by regional authorities. Although the recommended threshold of 30% absenteeism rate to trigger closure is high, in practice, many schools close each year during the annual seasonal influenza epidemics.

The other countries and administrative regions participating in this review do not routinely close schools during seasonal influenza epidemics. Some of these have relatively different uses of school closures to deal with public health crises. Notably HK has a considerable experience of closing schools during infectious disease outbreaks. For example, authorities in HK closed all schools during the SARS outbreak in 2003 [25] and all primary schools and kindergartens during a seasonal influenza outbreak in 2008 following two influenza-related deaths in children [20]. The NPI is usually accepted locally and indeed expected by the population including by politicians in HK. In other countries, it may be recognized that individual schools may choose to close temporarily for operational reasons (that is, if they have substantial student or staff absenteeism); but school closure seems to be used only rarely to manage infectious disease outbreaks [31]. In other countries, pre-scheduled school closures may coincide with the local seasonal influenza epidemics, which may have an unintentional impact on virus transmission. For example, South African schools were closed for the duration of the 2010 FIFA World Cup (a period of one month), which coincided with the seasonal influenza epidemic that year.

### School closures in national pandemic preparedness plans

All twelve countries and administrative regions discuss school closure as a mitigation measure during influenza pandemics; however, only three (Japan, Bulgaria and Thailand) indicate that they would certainly close schools during an influenza pandemic (Table 1). The other nine countries and administrative regions left the option open, stating it would depend on circumstances.

**Table 1:** Summary table

	**Bulgaria**	**China**	**England**	**France**	**Hong Kong**	**Italy**	**Japan**	**New York**	**New Zealand**	**Serbia**	**South Africa**	**Thailand**	**USA**
**Planning**													
Were school closures discussed in pre-pandemic plans prior to A(H1N1)pdm09?	Yes	Yes	Yes	Yes	Yes	Yes	Yes	Yes	Yes	Yes	Yes	Yes	Yes
Shall school closures be used according to pre-pandemic plans?	Yes	Maybe	Maybe	Maybe	Maybe	Maybe	Yes	Maybe	Maybe	Maybe	Maybe	Yes	Maybe
**Recommendations**	MoH for national closures. Local health authorities for local closures.	MoH and MoE	Advice given by Scientific Advisory Committee for Emergencies and Government Departments (Health, Children)	MoE led, with MoH and of Interior	MoH	MoH and MoE	MoE led, with MoH	NYC health authorities	MoH	MoH	MoE led, with MoH	MoH	CDC (in consultation with MoE and other partners)
Who made the recommendations about school closures during A(H1N1)pdm09?													
**Decision making**													
Who made the decision to close schools during A(H1N1)pdm09?	MoE for national closures. Local Education boards for local closures	Local government (MoH and MoE)	School headmaster in consultation with local public health officials	Local representative of State	Chief executive (equivalent of prime minister)	Local health authorities in agreement with the headmaster	Local authorities during initial phase. School principal later on	NYC health authorities in consultation with DoE for public schools; private schools made their own decision.	Local health authorities during containment period. School boards during mitigation period	MoE	Local government (local MoH and MoE)	School principal	Local/State Health Departments in collaboration with local school authorities
**Type of closure**													
School closure is standard policy during seasonal epidemics?	Yes	No	No	No	No	No	Yes	No	No	No	No	No	No
Were schools closed pro-actively during A(H1N1)pdm09?	Yes	No	No	No	Yes	No	Yes	No	No	Yes	No	No	No
Was there a policy of closing schools reactively?	Yes	Yes	No	Yes	Yes	Yes	Yes	Yes	Yes	Yes	No	Yes	Only from April 28th 2009 to May 5th 2009

MoE: Ministry of Education or equivalent; MoH: Ministry of Health or equivalent; DoE: Department of Education.

### National recommendations and local decisions during the A(H1N1)pdm09 pandemic

During the A(H1N1)pdm09 pandemic, the decision to close schools was a process that involved both national and local policy makers and school administrators. All countries and administrative regions made recommendations on school closure at the national level. There was often a lead agency/Ministry, which consulted with other agencies/Ministries to prepare recommendations.

In addition to recommendations made at the national level, recommendations were sometimes also made at a more regional (sub-national) level. This was, for example, the case in the US, South Africa and Bulgaria, where health is designated responsibility of the states/provinces.

For reactive closures, although recommendations were essentially made at the national level, decision making on school closure was always undertaken at the local level. In the UK and Thailand, closure was decided at the school level (by school principals, headmasters or school boards). In China, France and South Africa, the decision was made by local governments or local representatives of the state.

In some regions, decisions were made at different levels depending on the type of school or the pandemic phase. For example, in the US, different States and cities had different school closure policies, and the decision as to whether to close schools or not were being made locally. In New York City, the NYC Department of Health and Mental Hygiene made the decision for public schools in consultation with the Department of Education; but private schools made their decisions independently. In Japan, New Zealand and Thailand, regional policy makers were in charge of making the decision during the initial phase; but later on, closure was the responsibility of school officials.

#### Recommendations on school closure during the A(H1N1)pdm09 pandemic

During the A(H1N1)pdm09 pandemic, all countries and administrative regions included in this review acknowledged that some schools might have to close (or some classes to be dismissed) when high absenteeism of students/staff meant that the school could no longer function normally. All also implemented measures to reinforce infection control in schools (e.g., communication on hand hygiene, sick students/staff advised to stay home, etc). But the use of school closures to mitigate the pandemic varied substantially between locations.

#### School closure not recommended as a mitigation strategy early in the pandemic

Three countries (UK, US and South Africa) quickly decided not to recommend school closure to mitigate the pandemic at the national level. In the US, CDC advised on 28 April 2009 that dismissal of students for at least seven days should be strongly considered in schools with a confirmed or a suspected case epidemiologically linked to a confirmed case. However, the guidance was modified on 1 May 2009 recommending 14-day dismissals, but that modification was in effect for only four days which included a weekend. From 5 May 2009 onward, school dismissal was no longer recommended as a community mitigation measure in the US. In South Africa, a recommendation was made early during the onset of the local epidemic caused by A(H1N1)pdm09 (June 2009) not to use school closure as a mitigation strategy. In these three countries, the argument for not closing schools was that the pandemic was judged not to be severe and the potential benefits of school closure did not outweigh the deleterious socioeconomic impact that such an intervention would have.

In the European Union, in August 2009, a policy committee of Member States, chaired by the European Commission and advised by ECDC, issued a recommendation noting no reason to close schools proactively in Europe [32].

#### Reactive closures

All the other countries and administrative regions included in this analysis made recommendations for reactive school closure. The recommended strategies were usually proportionate, with, for example, closure of a class if more than a certain number of children were absent in the class and closure of the school if more than a certain number of classes were affected. Schools were usually recommended to close for at least seven days. As explained above, final decisions were often left to local or school authorities.

#### Pro-active closures

HK, Japan, Bulgaria and Serbia implemented pro-active school closures. HK and Japan did so early on in their spring 2009 wave, while Bulgaria and Serbia used it to mitigate their 2009 autumn waves.

In the early phase of the pandemic, HK implemented aggressive strategies to attempt to contain and later on to mitigate the spread the virus. Once the first case due to indigenous transmission was confirmed on 10 June 2009, they moved from a “containment phase” to a “mitigation phase” designed to relieve disease burden and mortality, primarily based on NPI [15,17]. The mitigation phase included: public health campaigns (improved hygiene, etc.), medical resource mobilization, opening of eight designated fever clinics (13 June 2009), and antiviral treatment of confirmed cases. In addition, there was an immediate proactive closure of kindergarten/primary schools (children up to 12) for at least 2 weeks (starting 12 June 2009) along with reactive closure of secondary schools with more than one confirmed case.

Osaka and Hyogo, two prefectures in Japan, implemented proactive school closures between 18-24 May 2009. In Osaka prefecture, at least 796 schools (270 high schools and 526 junior high schools) closed during that time period [33]; primary schools and kindergartens were also closed in some cities. In Hyogo prefecture, a total of 2,352 schools, kindergartens and universities closed during this time period. Pro-active closures also took place in whole/part of municipalities and school district between 18 May and 18 July 2009.

In Bulgaria, on 6 November 2009, the Ministry of Health declared a nationwide influenza epidemic and recommended to the Ministry of Education to close all schools in the country for five working days. Decisions on whether to close nurseries, child day care centers and the suspension of sessions at universities were delegated to the regional level.

In Serbia, a short 6-days school holiday (Thursday to Tuesday) was extended by 3 days nationwide during the first peak of the autumn pandemic wave in November 2009. In December, Christmas holidays were brought forward a week to mitigate a second peak.

### Extent of school closure

**Figure 3:**
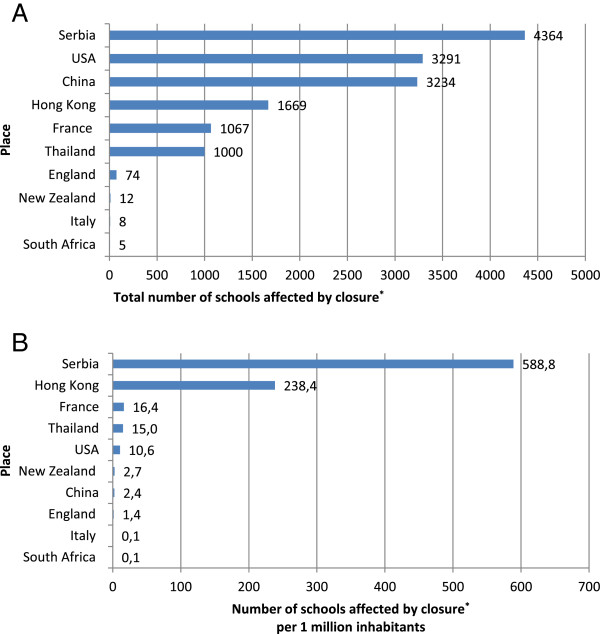
**Extent of school, grade and class closures in participating countries. A**: total number of schools affected by closures* during the 2009 H1N1pdm09 influenza pandemic; **B**: number of schools affected by closures during the 2009 H1N1pdm09 influenza pandemic per 1 million inhabitants. ( ^*^: e.g., may include complete school closure, grade closure and/or class closure).

For countries and administrative regions that provided information, Figure 3 shows the total number of schools and number of schools per 1 million inhabitants, which were affected by school, grade or class closures. Relative to population size, Serbia and HK, which closed all the schools, were by far the areas which implemented a school closure policy most widely. 160,742 closures were reported in Japan (1,258 reported closures per million inhabitants). No school closure count was available for Bulgaria, the last country that closed schools proactively. However, multiple reporting from the same school was frequent so that the Japanese figure is an over estimate (there are about 60,000 schools in Japan). Even so, it indicates that closures were on a massive scale in Japan.

**Figure 4:**
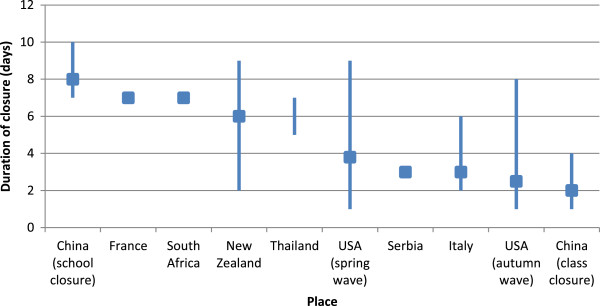
**Effective duration of closure (square: average; vertical line: range) in participating countries.** Countries are sorted by decreasing number of schools affected by closures.

It is interesting to note that although France, Thailand, China and Italy made relatively similar recommendations of closing schools reactively, the extent to which the policy was implemented locally varied markedly. For example from 0.13 (Italy) to 16 (France) schools affected per 1 million inhabitants though for those two countries there was no strong evidence of differences in impact of the pandemic [34]. For countries and administrative regions that provided information, the effective duration of closure was often shorter than the duration of seven days (or sometimes 14 days) recommended in many locations (Figure 4).

## Discussion

This review highlights the very diverse national and local experiences on school closures during the A(H1N1)pdm09 pandemic in eleven countries and one administrative region. It also showed the many challenges associated with the implementation of this intervention.

### Different school closure policies and expectations

First, there were important differences in the management of school closures across the participating countries. The processes (e.g., who was in charge of making recommendations, who was in charge of making the decision to close, etc) varied between countries. There were also marked differences in the school closure strategies with three countries and the European Union recommending to not use school closure as a mitigation strategy from relatively early on, while some countries recommended some sort of reactive closure, and still others implemented proactive closures at a large scale. Even among countries that made similar recommendations (e.g., allowing reactive closures) the actual extent of closure that took place varied substantially.

In the original plans of this research, we aimed to report on the reasoning behind each national policy decision to try to better understand why outcomes were so diverse. However, we quickly realized that this would be difficult to document in an objective way and that besides the outcome might actually provide limited insight. Indeed, the arguments in favor or against closure are already quite well known: it is a matter of finding the right balance between mitigating and delaying spread versus paying the potentially high cost associated with closure. Therefore, the question is not so much about the arguments used by countries to justify their decisions but more about why they made different appreciations of the health benefits and the economic and social costs of the intervention. In the end, we believe that the single most important factor to explain these differences was the very different public health practises and public expectations concerning school closures and influenza in the countries selected. For example, Japan and Bulgaria consider school closure as routine during seasonal influenza epidemics; and in HK, closures are expected from the population and politicians during large-scale infectious disease outbreaks. By contrast, it may require a severe pandemic for the intervention to be considered as a policy option, for example, in the UK. Obviously, these different interpretations were made possible because of the absence of a clear cut scientific/public health answer on the anticipated impact of school closures in a pandemic of moderate severity such as the A(H1N1)pdm09 pandemic, illustrated by the “grey zone” in Figure 1. Though closing schools was expected to have a significant impact on transmission dynamics in 2009 because of the age-specific transmission profile of the pandemic virus, one must ask if the benefits outweigh the high economic and social costs of closure, especially given that disease severity was relatively limited. This left considerable room for different views on the effectiveness to be transformed into policy. A last factor was the complexity of school systems and hence decision making with co-existing state and religious systems plus private schools in a number of countries.

### Monitoring and local decision making

Implementing reactive closures of schools on a relatively large scale requires that surveillance systems are in place in schools to monitor illness and absenteeism rates. In NYC, for example, the decision to close schools was based on trends in influenza like illness (ILI) visits to school nurses (sustained or sudden increase) and absenteeism. School health nurses who were in charge of gathering the data for their school were often overwhelmed so that the school data was generally not available for review until late afternoon and the decisions on closure could not take place until the evening - too late to inform parents.

Since school closures to mitigate influenza epidemics have been standard policy in Japan for many years, the country has developed an efficient system to monitor absenteeism in schools, and make decisions on closure on the basis of that system. The information about each school passed to relevant education boards (municipal board for most elementary and junior high schools and prefectural board for most high schools), and directly to the Ministry of Education for most private schools. The information is analyzed at the Ministry of Education and shared with the Ministry of Health. This process is performed on a daily basis. In contrast, pro-active closures as implemented in Osaka and Hyogo prefectures in Japan or in HK were simply triggered by the first local pandemic cases that were not linked to importation.

### Communication challenges

School closure was associated with a range of communication challenges.

#### With parents and school staff

Good communication with parents and school staff is an important part of the control strategy and may require substantial effort. For example, in New York City, some of the communication challenges with parents and school staff included:

• **Translating:** By local regulations, all parental communications had to be translated into nine languages.

• **Notifying closures:** In the spring 2009 wave, since decisions of closures had to be made after the end of the school day, notifications of closure had to be made via media and word of mouth. Representatives of the Department of Health and of the Department of Education were also present in the morning of closure at school to notify or discuss with parents.

• **Making parents part of the strategy:** In the autumn 2009 wave, the Department of Education did not plan to close schools with influenza activity but rather emphasized teaching students preventive measures (e.g., wash/sanitize hands often, avoid touching mouth and nose etc) and parents were instructed to keep children home if they had ILI. Communication with families and principals was therefore an essential part of the strategy: letters were sent home to families with the children during the first week of school and throughout the influenza season; information materials were provided to schools; outreach was implemented to community education councils and parent groups; weekly influenza updates were sent to all school principals and there was also ongoing communication with school nurses. At the end of each school day, the Department of Education reported online attendance rates and instances of ILI in public schools. If a school reported five cases of ILI among students in attendance, parents were sent a second letter on keeping ill children at home and the school reinforced messages on hand washing and covering coughs.

• **Dealing with staff concerns:** To address the concerns of school staff about their safety, the city developed an Influenza Health and Safety Program.

#### National to local communications & communications between agencies

Managing the pandemic and school closures often required very close interactions between the different agencies and the different levels of policy making.

Early in the pandemic, in the US, CDC had daily conference calls with Public Health State officials where the management of the pandemic, including school closure policies, was discussed. Those conference calls were critical for sharing data, discussing policy questions and recommendations. The prompt change of CDC recommendations on school closure (between 28 April and 5 May 2009) although primarily based on the accumulated epidemiologic data from the US outbreaks was also corroborated by the feedback that CDC received during those conference calls, where some state officials regarded the guidance as overly disruptive for the locally perceived level of severity. Similar close communication between agencies was also reported in a number of the other countries.

There were sometimes differences in the perception of risk between the national level (at which most of recommendations were made) and the local level (where decision making took place). For example, in France, it was reported that the perception of risk was higher at the national level where potential deaths among children was seen as an incentive to act preventively and in a context where public health issues are interpreted in terms of political responsibilities by major ministries. Also there had been considerable planning on how to educate and manage children if the schools had to close. But local authorities were reluctant to close schools due to a combination of lower risk perception and a general pattern of local public policy-making where the primary objective is to maintain normal life, unless an immediate and major risk is identified. There were also doubts at the local level about the overall strategy given its sophistication. Parents who had a low perception of the risk considered the intervention as a constraint. Nevertheless there were significant numbers of school closures in France.

#### Communicating in a context of uncertainty

The pandemic also highlighted the difficulty in communicating in a context of uncertainty and where risk assessment may quickly change. For example, in many countries, there was sporadic media criticism of rapidly changing guidance, and differences in practice between localities and over time in spite of explicit statements in the initial guidance that changes in guidance would be forthcoming pending more data.

### Authority to close schools

One of the challenges faced when managing school closures was that different schools systems (e.g., public, private and parochial) often coexist. For example in NYC the public (state) system is centrally operated and closures in that system were decided by the Chancellor or the like; but that is not the case of the private and parochial systems for which closures were done at the discretion of the school.

In many countries, the national government could impose nationwide closures. But this is not necessarily straightforward. For example, in semi-autonomous or federal countries like South Africa or the USA, each state/province has the authority to make their own decision even if this means a seeming inconsistency across the country.

### Limitations

The countries and administrative regions participating in this review are not a representative sample of countries around the world. Indeed, our intention was not to make a systematic review but to describe the diversity of school closure experiences and provide examples from national and local perspectives. Therefore it is not possible to generalize on the extent of school closure around the world from this work.

In this review, we described national and local experiences on school closure. We presented the various policy processes leading to closure (from the elaboration of recommendations to the implementation of the policy) as well as the extent of closure (e.g., how many schools closed and for how long). We also discussed policy challenges associated with the intervention. However, important areas of research on school closures, such as the impact on spread and health care provision as well as the economical and social cost of closing schools, were left out of the review. A review of those aspects of school closure can be found in [12].

## Conclusions

Even in the relatively mild severity scenario of the A(H1N1)pdm09 pandemic [35], the findings of this work indicate that there was a range of responses and applications of the school closures policies. The processes including who was in charge of making recommendations and who was in charge of making the decision to close, the school-based control strategies, the extent of school closures, the public health tradition of responses and expectations on school closure varied greatly between countries.

Consistent to the experience from earlier influenza pandemics [7-11], the A(H1N1)pdm09 pandemic confirmed the impact that some forms of school closing could have on the community-wide influenza transmission dynamics [13-17]. Given that epidemiologic evidence and looking very practically at pressures exerted on policy makers during an emerging pandemic, it is reasonable to predict that the school closure policy will be considered and implemented in future pandemics, at an extent that will depend on the perceived severity and perceived impact of the pandemic virus on children’s health. There is, therefore, good reason for countries to persist with working on the school closure policies and assessing their effect on transmission and overall societal impact. This review has demonstrated that much work remains to ensure smooth implementation of the school closure policy, a feasible NPI that may be used during future influenza pandemics (Table 2).

**Table 2:** Recommended areas for further work on school closures by authorities

**Area**	**Description**
**Pandemic planning**	Ensure that school closures (as a public health intervention or due to large absenteeism in schools) is included in generic pandemic planning.
**Triggers**	Agree on triggers for proactive and reactive closures in a pandemic and how they would be operated at the local level.
**National decision making**	Prepare arrangements for national decision-making on school closures and how adjoining counties would apply these.
**Mitigating adverse effects**	Develop arrangements for mitigating the adverse impact of school closures notably for alternative care arrangements and continuing education.
**Special schools**	Consider how special schools would be included in these arrangements.
**Communication**	Develop communication plans and materials for school staff, parents and the media.
**Local planning**	Ensure there are robust local plans for closures across complex school systems and exercise these plans on occasions for pandemic and other emergencies (such as extreme weather).

## Competing interests

SC received consulting fees from Sanofi Pasteur MSD. BJC received research funding from MedImmune Inc. and Sanofi Pasteur, and consults for Crucell NV. There are no other competing interests to declare.

## Authors’ contributions

SC, MDVK, AN designed the study; BNA, MC, BJC, PG, DH, MK, PK, KU, HO, AP, CR, GS, TS, AU, CW, IW, HY shared their local and national experience and provided data; SC, MDVK analysed data; All authors wrote paper. All authors read and approved the final manuscript.

## Pre-publication history

The pre-publication history for this paper can be accessed here:

http://www.biomedcentral.com/1471-2334/14/207/prepub

## Supplementary Material

Additional file 1 Data collection form. Click here for file
